# Acute adrenal insufficiency following arthroplasty: a case report and review of the literature

**DOI:** 10.1186/1756-0500-6-370

**Published:** 2013-09-12

**Authors:** Stylianos Mandanas, Maria Boudina, Alexandra Chrisoulidou, Katerina Xinou, Efterpi Margaritidou, Spyros Gerou, Kalliopi Pazaitou-Panayiotou

**Affiliations:** 1 Department of Endocrinology, Theagenion Cancer Hospital, Al. Simeonidi st, Thessaloniki 54007, Greece

**Keywords:** Adrenal insufficiency, Arthroplasty, Heparin

## Abstract

**Background:**

Acute adrenal insufficiency is a potentially lethal condition rarely caused by bilateral adrenal haemorrhage due to heparin use. Most of the times, it is difficult to establish the diagnosis, as symptoms are not specific. Few cases have been reported in the literature.

**Case presentation:**

A 52-year-old Caucasian woman presented with abdominal pain, vomiting and weakness nine days after arthroplasty and heparin use. Hyperkalemia, low cortisol and high adrenocorticotropic hormone levels were found, indicating adrenal insufficiency. Magnetic resonance imaging of the upper abdomen was compatible with preceding adrenal haemorrhage. Hydrocortisone and fludrocortisone were administered. Review of the literature revealed 36 cases of postoperative adrenal haemorrhage which are presented briefly.

**Conclusion:**

Postoperative acute adrenal insufficiency due to haemorrhage is a rare condition. If patients are treated based on clinical suspicion, they have good chances to survive. Hydrocortisone is given permanently in the majority of the patients.

## Background

Acute adrenal failure (AAF) is a potentially life-threatening complication presenting with non-specific symptoms as abdominal pain, nausea, fever, tachycardia, hypotension and lethargy [1]. However, hyponatremia and hyperkalemia as indicators of adrenal insufficiency should be evaluated very thoroughly [2].

AAF may occur in patients with previously undiagnosed primary adrenal insufficiency and sometimes after bilateral adrenal infarction or postoperative haemorrhage in otherwise healthy individuals [1]. The postoperative period carries a high risk for haemorrhage, as platelet consumption may lead to bleeding in vital organs, independently of anticoagulant administration. Additionally, the use of heparin represents an independent risk factor that predisposes to haemorrhage [3]. Injuries during procedures such as extracorporeal shock-wave lithotripsy or electroconvulsive therapy [4,5] or use of certain materials and techniques may also predispose to coagulopathy and haemorrhage [6]. Moreover, hypothermia itself may play an important role in bleeding [6]. As signs and symptoms are not specific, they may easily lead to wrong diagnosis, such as postoperative septic shock or inflammation.

Total hip arthroplasty is a common surgical procedure associated with deep venous thrombosis and pulmonary embolism that are prevented by the use of anticoagulants [7,8]. The extensive use of anticoagulants in these operations explains the fact that bilateral adrenal haemorrhage is more often observed after knee or hip arthroplasty. However, orthopaedic surgery is probably associated with risk factors (other than anticoagulants) which lead to haemorrhage [9].

The use of low molecular weight heparin (LMWH) to avoid postoperative thromboembolic events can induce thrombocytopenia (heparin-induced thrombocytopenia [HIT]) and may lead to bilateral adrenal haemorrhage [2]. A pre-test clinical score (including thrombocytopenia, timing of platelet count fall, thrombosis, presence of other causes for thrombocytopenia, the so-called 4 T's) has been developed to establish the clinical suspicion of HIT [10], while heparin-platelet factor 4-immunoglobulin G (IgG) (PF4-IgG) antibodies and 14C-serotonin release assay are useful diagnostic tests [2].

The incidence of bilateral adrenal haemorrhage is estimated to be around 4.7–6.2 cases per million habitants in developed countries, but the prevalence is much higher in hospitalized patients, arising to 1.1% of them [11,12]. There are difficulties in establishing an early diagnosis of the disease [13] and adrenal haemorrhage is usually a post-mortem finding during autopsy performed to unravel the cause of death.

In addition to the elusive clinical presentation, imaging may also be deceiving: the enlarged haemorrhagic adrenals may be misdiagnosed as neoplastic masses, with irregular margins, though maintain their adreniform shape. Acute and subacute adrenal haemorrhage show high attenuation (50–90 HU) at unenhanced computed tomography (CT), without enhancement following intravenous (IV) contrast. In doubtful cases, decreased density and size of the adrenals during follow-up as well as the presence of calcifications may be extremely useful to confirm the diagnosis. Magnetic resonance imaging (MRI) is the most sensitive and specific imaging modality to confirm adrenal haemorrhage. Like CT, the appearance of adrenal haemorrhage on MRI also depends on the progression of the bleeding. The most characteristic sign on MRI is the low signal ring on T2 sequences during the chronic phase [14].

The aim of the present work was a) to present a case of acute adrenal insufficiency caused by bilateral adrenal haemorrhage observed after arthroplasty and b) to summarize the published data regarding this rare and interesting clinical entity.

Computerized literature search was performed in the PubMed electronic database. The original query provided 71 possibly relevant articles. Furthermore, 10 articles were retrieved after searching the “Related Articles” link and the references. Of these, 34 were finally selected (including 36 case reports), whereas the remaining 47 articles were excluded for the following reasons: spontaneous bilateral adrenal haemorrhage without apparent cause (n=9); concurrent diseases (n=7); bilateral adrenal haemorrhage due to heparin for other reason, not postoperatively (n=27); article in Japanese (n=1); reviews and letters to the editor (n=3).

In summary, 36 cases of postoperative bilateral adrenal haemorrhage have been documented. Mean age of patients during haemorrhage was 65.2 years (range 44–83) and there was no particular sex distribution. Abdominal pain, fever, vomiting and hypotension were the main symptoms at presentation, usually occurring between first and second week after surgery. Hyponatremia and hyperkalemia were the most common laboratory findings. In 27 out of 36 patients, diagnosis was made by CT scan, in two by abdominal ultrasound and in one by exploratory laparotomy. In four patients the diagnosis was confirmed after their death during autopsy and in two cases the imaging was not described. Nine patients succumbed to adrenal insufficiency.

Patients’ clinical characteristics and diagnostic methods are summarized in Tables 1 and 2. Reported cases are divided into two groups: the first includes 21 patients with adrenal insufficiency after orthopaedic surgery (Table 1) and the second reports 15 patients with adrenal insufficiency after any other surgery (Table 2).

**Table 1:** A**drenal insufficiency after orthopaedic surgery**

**Cases**	**Author**	**Country**	**Age**	**Sex**	**Type of surgery**	**Diagnosis**	**Symptoms**	**Biochemical exams**
1	Findling et al., 1987 [15]	USA	44	M	unspecified	CT	abdominal pain, vomiting, hypotension	hyponatremia, hyperkalemia
2	Delhumeau et al., 1989 [16]	France	76	F	total hip arthroplasty	abdominal ultrasound	abdominal pain, fever, hypotension, altered consciousness	hyponatremia, natriuresis, thrombocytopenia
3			74	M	tibial osteosynthesis	not done	abdominal pain, fever, hypotension, shock, altered consciousness	hyponatremia, thrombocytopenia
4			62	M	tibial osteosynthesis	CT	abdominal pain, asthenia, nausea	hyponatremia, thrombocytopenia
5	Ernest and Fischer, 1991 [17]	Australia	68	F	total hip arthroplasty	CT	fever, hypotension	hyponatremia, hyperkalemia
6	Souied et al., 1991 [18]	France	63	F	total hip arthroplasty	CT	fever, hypotension	hyponatremia, hyperkalemia
7	Bleasel et al., 1992 [19]	Australia	69	F	total knee arthroplasty	CT	fever, nausea, vomiting, abdominal pain, hypotension	hyponatremia, hyperkalemia
8	Hardwicke and Kisly, 1992 [20]	USA	63	F	bilateral total knee arthroplasty	CT	fever, nausea, anorexia, vomiting, abdominal pain, confusion, feeling of illness, hypotension	anemia, hyponatremia, hyperkalemia
9	Delhumeau et al., 1993 [21]	France	74	M	total hip arthroplasty, thrombectomy of both limbs due to bilateral arterial thrombosis	CT	abdominal pain, fever, hypotension, abdominal tenderness	hyponatremia, hyperkalemia
10	Santonastaso et al., 1993 [22]	Italy	not reported	F	osteotomy	CT	somnolence, asthenia, hypotension	not reported
11	Ries et al., 1994 [23]	USA	61	F	bilateral total knee arthroplasty	autopsy	abdominal discomfort, nausea, collapse	none
12	Cozzolino et al., 1997 [24]	USA	66	F	total knee arthroplasty	CT	nausea, anorexia, emesis, lethargy	hyponatremia, hyperkalemia, anemia, azotemia
13	Rowland et al., 1999 [25]	Australia	50	M	unspecified	CT	fever, abdominal pain, dizziness, hypotension	hyponatremia
14	Caubet et al., 1999 [26]	France	63	F	tibial osteosynthesis	CT	fever, hypotension, tachypnea	thrombocytopenia, hyponatremia, hyperkalemia, elevated INR
15	Scheffold et al., 2001 [27]	Germany	63	M	intracondylar nail-extension	abdominal ultrasound	abdominal pain, fever	leukocytosis
16	LaBan et al., 2003 [28]	USA	82	F	bilateral knee arthroplasty	CT	abdominal pain, nausea	elevated INR, hyponatremia
17	Schuchmann et al., 2005 [8]	USA	83	F	bilateral total knee arthroplasty	autopsy	anxiety, hypertension, midback pain, shortness of breath, shock	hyponatremia, hypochloremia, leukocytosis, anemia, elevated INR
18	Kurtz and Yang, 2007 [29]	USA	54	F	total hip arthroplasty	CT	fever, abdominal pain, orthostatic syncope	not reported
19	Mongardon et al., 2007 [30]	France	64	M	total hip arthroplasty	CT	fever, abdominal pain, confusion, hypotension	thrombocytopenia, high urinary sodium
20	Thota et al., 2012 [31]	USA	68	M	bilateral total knee arthroplasty	CT	fever, abdominal discomfort, anorexia, fatigue, hypertension, tachycardia, altered mental status	hyperglycemia, leukocytosis, anemia, thrombocytopenia, hyponatremia
21	Chow et al., 2012 [32]	USA	44	M	bilateral total knee arthroplasty	CT	hypotension, abdominal pain, tachycardia, fever	leukocytosis, D-dimers elevation, hyponatremia, thrombocytopenia

**Table 2:** A**drenal insufficiency after any surgery except for orthopaedic surgery**

**Cases**	**Author**	**Country**	**Age**	**Sex**	**Type of surgery**	**Diagnosis**	**Symptoms**	**Lab results**
1	Steer and Fromm, 1980 [33]	USA	71	M	cholecystectomy	not done	abdominal pain, fever, vomiting, anorexia, weakness, hypotension	eosinophilia, hyponatremia, hyperkalemia, prothrombin time elevation
2	Jacobson et al., 1986 [34]	USA	55	M	retropubic prostatectomy	CT	fever, tachycardia, hypotension, dyspnea, abdominal pain, nausea, vomiting, diarrhea, ileus	hyponatremia, hyperkalemia
3	Miller et al., 1986 [35]	USA	81	F	aorto-iliac aneurysmectomy	CT	fever, nausea, vomiting, abdominal pain, lethargy	hyponatremia, hyperkalemia, leukocytosis
4	Homcy and Southern, 1989 [36]	USA	71	M	colectomy	autopsy	confusion, hypotension	hyponatremia
5	Ting et al., 1992 [37]	USA	62	M	coronary bypass grafting	CT	left flank pain, fever, tachypnea, tachycardia, tired, hypotension	leukocytosis, hyponatremia, hyperkalemia
6	Leschi et al., 1994 [38]	France	63	M	aortofemoral bypass graft	autopsy	confusion	hyponatremia, hyperkalemia
7	Belmore and Walters, 1995 [39]	USA	53	M	laparoscopic cholecystectomy	CT	abdominal pain, anorexia, fatigue, hypotension, dehydration	hyponatremia, hyperkalemia, hyperamylasemia, partial thromboplastin time prolonged
8	Scheiwiller et al., 2002 [40]	Switzerland	71	M	low anterior rectum resection	CT	anorexia, abdominal pain, fever, hypotension	hyponatremia
9	Sousa Escandon et al., 2002 [41]	Spain	82	M	partial nephrectomy for renal adenocarcinoma	CT	abdominal pain, nausea, fever, hypotension, severe respiratory distress	hyponatremia, azotemia, anemia, leukocytosis, thrombocytosis
10	Bakaeen et al., 2005 [42]	USA	51	M	coronary artery bypass graft	CT	fever, abdominal pain, diarrhea, orthostatic hypotension, tachycardia	thrombocytopenia
11	Gutenberg et al., 2007 [43]	Germany	69	M	intracranial tumor surgery	exploratory laparotomy	hypotension, abdomen tense	not reported
12	Munoz Corsini et al., 2008 [44]	Spain	63	M	right hemicolectomy	CT	disorientation, difficulty in breathing, tachycardia, fever	leukocytosis, anemia, increase in fibrinogen,
13	Peel and Whitelaw, 2011 [45]	USA	60	M	right hemicolectomy	CT	backache, fever, hypotension, agitation, confusion	hyponatremia, thrombocytopenia, leukocytosis
14	Rosenberger et al., 2011 [2]	USA	69	M	gastrectomy, esophagojejuno- stomy, cholecystectomy	CT	abdominal and flank pain, tachypnea, tachycardia, hypotension, oliguria, myocardial infarction	oliguric renal failure
15	Balsach Sole et al., 2012 [46]	Spain	70	M	cephalic duodenopancreatectomy	CT	lethargy, hypotension	hyponatremia, hypoglycemia

## Case presentation

### Patients’ history

A 52-year-old Caucasian woman, mother of two children, underwent right hip arthroplasty and was administered LMWH (enoxaparin 8,000 IU per day) for seven days in order to prevent thromboembolic events. Arthroplasty was successfully completed without intraoperative or early postoperative complications, except for a fall in platelet count from 214,000/μL to 116,000/μL. Haemoglobin and white blood cell count were normal. The patient was discharged from the hospital in good condition without any sign or symptom of haemorrhage, thromboembolism or infection.

### 9th postoperative day, emergency department

On postoperative day 9, she presented in the emergency department complaining of abdominal pain, vomiting and weakness. She was dehydrated and tachycardic (105 beats per minute). Decreased skin turgor and low blood pressure (90/60mm Hg) were observed. Biochemical exams indicated hyponatremia (128mmol/L, normal range 136–145) and hyperkalemia (5.97mmol/L, normal range 3.5-5.1), normal serum glucose levels, as well as normal kidney and liver function. The patient was afebrile and the wound healed satisfactory. The administration of isotonic solutions was decided but the patient responded poorly. Intravenous use of dopamine was added thereafter, resulting in slight improvement of clinical symptoms, mainly vomiting and arterial blood pressure. As the abdominal pain was persistent, the patient underwent abdominal CT which was indicative of “bilateral adrenal adenomas”. Due to this finding, the patient was referred to our department for further evaluation.

### Referral to the endocrinology department

**Figure 1:**
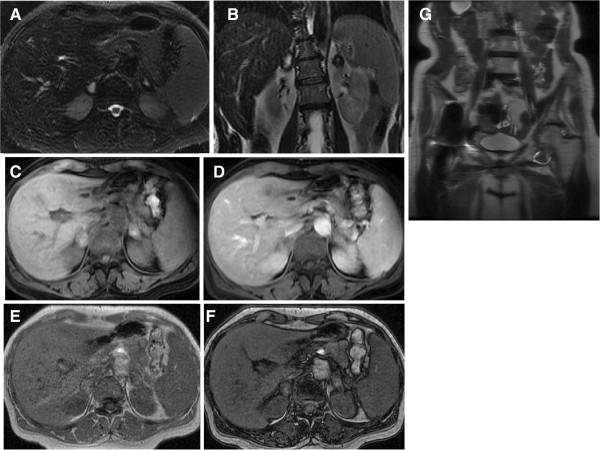
**Abdominal magnetic resonance imaging obtained 15 days after surgery.** Axial **(A)** T2 weighted image with fat suppression, coronal **(B)** T2 weighted image, in and out of phase axial **(C**, **D)** T1 images, axial **(E**, **F)** T1 weighted images with fat saturation before and after contrast administration, coronal **(G)** T2-weighted image. Bilateral adrenal lesions showing high signal both on T1-weighted **(E)** and on T2-weighted images **(A**, **B)**, and low signal rim on T2-weighted images **(A**, **B)**, representing haemosiderin. The lesions do not show any signal drop-off on T1 in- **(C)** and out- of phase images **(D)**. After intravenous contrast administration **(F)** no enhancement is depicted. These findings are consistent with chronic adrenal hematomas. Note the signal void in the right pelvis due to the presence of a metallic right hip prosthesis.

Considering the clinical signs of dehydration and the presence of hyperkalemia (5.72mmol/L, normal range 3.5-5.1) and hyponatremia (130mmol/L, normal range 136–145), adrenal insufficiency was suspected. Cortisol levels were measured and found to be very low (cortisol 40nmol/l, normal range 70-250nmol/l). A 250-μg adrenocorticotropic hormone (ACTH) stimulation test (Synacthen test) was performed for further evaluation; no increase in cortisol was observed confirming the diagnosis of primary adrenal insufficiency (basal cortisol equal to 32.9nmol/l, 30 and 60 min after Synacthen equal to 34nmol/l and 32.1nmol/l respectively, normal range 70-250nmol/l). ACTH levels were very high (1763.4pg/ml, normal range 9–52). MRI of the upper abdomen showed the presence of bilateral adrenal “lesions” with greatest dimension 2.3cm on the right and 2.5cm on the left. On T2 weighted-images the above mentioned findings had high signal intensity with a low signal intensity ring along the periphery compatible with the presence of haemosiderin consequence of previous haemorrhage and chronic hematomas (Figure 1). No signal drop-off on in- and out- of phase images and no significant enhancement following iv contrast was depicted.

### Diagnostic evaluation and therapy

Considering the variety of causes that could trigger bilateral adrenal haemorrhage in accordance with the medical history of the patient, traumatic injury, burns or pregnancy were ruled out [13]. Furthermore, she was afebrile without any clinical signs of septic shock. Antiphospholipid syndrome was excluded due to the absence of vascular thrombosis and the history of two normal deliveries. Based on the fact that the patient had received LMWH postoperatively, haemorrhage was considered to be the cause of adrenal failure. Heparin-PF4-IgG antibodies (measured by enzyme-linked immunosorbent assay [ELISA]) were negative. Moreover, the low pretest clinical score for HIT (total 2 points, 1 point from thrombocytopenia and 1 point from surgery) in the patient was correlated with high- negative predictive value for heparin induced adrenal haemorrhage [10].

**Figure 2:**
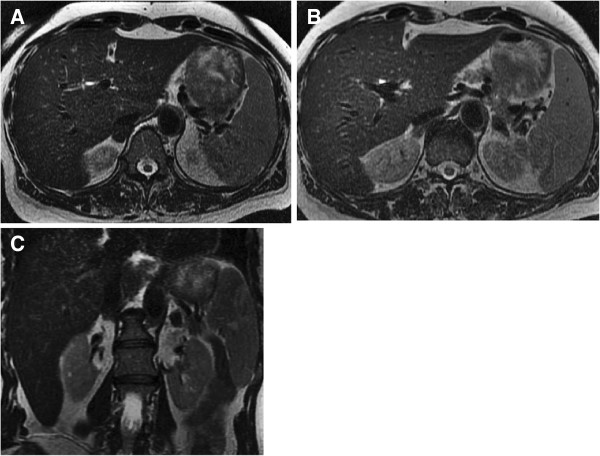
**Abdominal magnetic resonance imaging obtained 3.5 months after surgery.** Axial **(A**, **B)** and coronal **(C)** T2-weighted images showing a decrease in size and a change of signal of the bilateral adrenal hematomas. The lesions now have homogeneous low signal, due to haemosiderin.

Replacement therapy with hydrocortisone and fludrocortisone was started. Three months later, ACTH levels fell to 397pg/ml. Adrenal MRI showed that the lesions had decreased in size and had homogeneous low signal on T2 weighted images, findings consistent with the evolution of hematomas (Figure 2). The oral replacement with hydrocortisone and fludrocortisone remains until the present time.

## Conclusions

Bilateral adrenal haemorrhage is a rare disease which can follow major surgical operations. It should be suspected in patients presenting with fever, abdominal pain, confusion and hemodynamic collapse not responding to standard medical treatment [31]. The increased incidence after orthopaedic surgery, the association with anticoagulants use and the great mortality in misdiagnosed cases should keep physicians alerted.

## Consent

Written informed consent was obtained from the patient for publication of this Case Report and any accompanying images. A copy of the written consent is available for review by the Editor-in-Chief of this journal.

## Abbreviations

AAF: Acute adrenal failure; LMWH: Low molecular weight heparin; HIT: Heparin induced thrombocytopenia; IgG: Immunoglobulin G; PF4-IgG: Platelet factor 4- immunoglobulin G; CT: Computed tomography; IV: Intravenous; MRI: Magnetic resonance imaging; ACTH: Adrenocorticotropic hormone; ELISA: Enzyme-linked immunosorbent assay.

## Competing interests

The authors declare that they have no competing interests.

## Authors’ contributions

SM and MB reviewed the literature and prepared the draft. KX performed and discussed imaging. EM collected patients’ clinical data. SG performed the immunoassay. AC and KPP conceived the idea and made the amendments of the manuscript. All authors read and approved the final manuscript.

## Disclosure statement

The authors have nothing to disclose.

## References

[B1] Bouillon R Acute adrenal insufficiency Endocrinol Metab Clin North Am 2006 35 767 775 10.1016/j.ecl.2006.09.004 17127145

[B2] Rosenberger LH Smith PW Sawyer RG Hanks JB Adams RB Hedrick TL Bilateral adrenal hemorrhage: the unrecognized cause of hemodynamic collapse associated with heparin-induced thrombocytopenia Crit Care Med 2011 39 833 838 10.1097/CCM.0b013e318206d0eb 21242799 PMC3101312

[B3] Chang JC Review: Postoperative thrombocytopenia: with etiologic, diagnostic, and therapeutic consideration Am J Med Sci 1996 311 96 105 10.1097/00000441-199602000-00009 8615383

[B4] Donald IP Freeman CP Adrenal hemorrhagic necrosis following electroconvulsive therapy Lancet 1982 31 277 6124705 10.1016/s0140-6736(82)90363-4

[B5] Lai YL Chang WC Huang HH Obscure abdominal pain in a 55-year-old man. Diagnosis: Intra-abdominal hemorrhage with adrenal hematoma. Gastroenterology 2010 139 387 10.1053/j.gastro.2009.09.066 20600068

[B6] McKenna R Abnormal coagulation in the postoperative period contributing to excessive bleeding Med Clin North Am 2001 85 1277 1310 10.1016/S0025-7125(05)70378-3 11565500

[B7] Geerts WH Prevention of venous thromboembolism: American College of Chest Physicians Evidence-Based Clinical Practice Guidelines (8th Edition) Chest 2008 133 Suppl 6 381 453 10.1378/chest.08-0656 18574271

[B8] Schuchmann JA Friedman PA Bilateral adrenal hemorrhage: an unusual complication after bilateral total knee arthroplasty Am J Phys Med Rehabil 2005 84 899 903 10.1097/01.phm.0000176351.97994.73 16244529

[B9] Oberweis BS Nukala S Rosenberg A Guo Y Stuchin S Radford MJ Berger JS Thrombotic and bleeding complications after orthopedic surgery Am Heart J 2013 165 427 433 10.1016/j.ahj.2012.11.005 23453114 PMC3595114

[B10] Lo GK Juhl D Warkentin TE Sigouin CS Eichler P Greinacher A Evaluation of pretest clinical score (4 T's) for the diagnosis of heparin-induced thrombocytopenia in two clinical settings J Thromb Haemost 2006 4 759 765 10.1111/j.1538-7836.2006.01787.x 16634744

[B11] Bharucha T Broderick C Easom N Roberts C Moore D Bilateral adrenal haemorrhage presenting as epigastric and back pain JRSM Short Rep 2012 3 15 10.1258/shorts.2011.011107 22479678 PMC3318239

[B12] Vella A Nippoldt TB Morris JC 3rd Adrenal hemorrhage: a 25-year experience at the Mayo Clinic Mayo Clin Proc 2001 76 161 168 11213304 10.1016/S0025-6196(11)63123-6

[B13] Arlt W Allolio B Adrenal insufficiency Lancet 2003 361 1881 1893 10.1016/S0140-6736(03)13492-7 12788587

[B14] Wah TM Guthrie JA Joyce AD De la Rosette JJMCH, Manyak MJ, Harisinghani MG, Wijkstra H Cross-Sectional Imaging of adrenal masses Imaging in Oncological Urology 2009 London: Springer 5 27

[B15] Findling JW Korducki JM Lahiri PK Miller DD Raff H Bilateral adrenal hemorrhage associated with heparin-induced thrombocytopenia Wis Med J 1987 86 27 29 3564517

[B16] Delhumeau A Houet JF Bourrier P Bukowski JG Granry JC Heparin-induced thrombocytopenia complicated by hematoma of the adrenal glands and acute adrenal insufficiency Ann Fr Anesth Reanim 1989 8 656 658 10.1016/S0750-7658(89)80183-2 2633663

[B17] Ernest D Fisher MM Heparin-induced thrombocytopaenia complicated by bilateral adrenal haemorrhage Intensive Care Med 1991 17 238 240 10.1007/BF01709885 1744311

[B18] Souied F Pourriat JL Le Roux G Hoang P Kemeny JL Cupa M Adrenal hemorrhagic necrosis related to heparin-associated thrombocytopenia Crit Care Med 1991 19 297 299 10.1097/00003246-199102000-00033 1842892

[B19] Bleasel JF Rasko JE Rickard KA Richards G Acute adrenal insufficiency secondary to heparin-induced thrombocytopenia-thrombosis syndrome Med J Aust 1992 157 192 193 1635495 10.5694/j.1326-5377.1992.tb137086.x

[B20] Hardwicke MB Kisly A Prophylactic subcutaneous heparin therapy as a cause of bilateral adrenal hemorrhage Arch Intern Med 1992 152 845 847 10.1001/archinte.1992.00400160133026 1558445

[B21] Delhumeau A Moreau X Chapotte C Houi N Bigorgne JC Heparin-associated thrombocytopenia syndrome: an underestimated etiology of adrenal hemorrhage Intensive Care Med 1993 19 475 477 10.1007/BF01711091 8294632

[B22] Santonastaso M Bovo P Colaceci R Corbanese U Ruga P Acute adrenal failure due to adrenal hemorrhagic necrosis secondary to heparin-induced thrombocytopenia Recenti Prog Med 1993 84 687 690 8235035

[B23] Ries MD Guiney W Jr Lynch F Fatal massive adrenal hemorrhage after bilateral total knee arthroplasty J Arthroplasty 1994 9 559 562 10.1016/0883-5403(94)90106-6 7807117

[B24] Cozzolino D Peerzada J Heaney JA Adrenal insufficiency from bilateral adrenal hemorrhage after total knee replacement surgery Urology 1997 50 125 127 10.1016/S0090-4295(97)00102-7 9218034

[B25] Rowland CH Woodford PA De Lisle-Hammond J Nair B Heparin-induced thrombocytopenia-thrombosis syndrome and bilateral adrenal haemorrhage after prophylactic heparin use Aust N Z J Med 1999 29 741 742 10.1111/j.1445-5994.1999.tb01626.x 10630659

[B26] Caubet O Pillet O Cherifi A Mayet T Castaing Y Favarel Garrigues JC Acute adrenal insufficiency due to bilateral adrenal hematoma following severe thrombopenia induced by low-molecular-weight heparin Presse Med 1999 28 1010 1012 10379347

[B27] Scheffold N Schöngart H Berentelg J Prager P Cyran J Rare complication of a heparin-induced thrombocytopenia type II Dtsch Med Wochenschr 2001 126 329 333 10.1055/s-2001-12094 11305201

[B28] LaBan MM Whitmore CE Taylor RS Bilateral adrenal hemorrhage after anticoagulation prophylaxis for bilateral knee arthroplasty Am J Phys Med Rehabil 2003 82 418 420 12704285 10.1097/01.PHM.0000064741.97586.E4

[B29] Kurtz LE Yang S Bilateral adrenal hemorrhage associated with heparin induced thrombocytopenia Am J Hematol 2007 82 493 494 10.1002/ajh.20884 17266058

[B30] Mongardon N Bruneel F Henry Lagarrigue M Legriel S Revault D'Allonnes L Guezennec P Troche G Bedos JP Shock during heparin-induced thrombocytopenia: look for adrenal insufficiency Intensive Care Med 2007 33 547 548 10.1007/s00134-006-0487-9 17186288

[B31] Thota R Porter J Ganti AK Peters E Hemodynamic collapse following bilateral knee arthroplasty: a mysterious case J Thromb Thrombolysis 2012 33 3 5 10.1007/s11239-011-0640-3 21938456

[B32] Chow VW Abnousi F Huddleston JI Lin LH Heparin-induced thrombocytopenia after total knee arthroplasty, with subsequent adrenal hemorrhage J Arthroplasty 2012 27 15 18 1413.e 10.1016/j.arth.2011.02.016 22397862

[B33] Steer M Fromm D Recognition of adrenal insufficiency in the postoperative patient Am J Surg 1980 139 443 446 10.1016/0002-9610(80)90312-8 6244751

[B34] Jacobson SA Blute RD Jr Green DF McPhedran P Weiss RM Lytton B Acute adrenal insufficiency as a complication of urological surgery J Urol 1986 135 337 340 3944871 10.1016/s0022-5347(17)45632-1

[B35] Miller EH Woldenberg DH Gittler RD Zumoff B Bilateral adrenal hemorrhage following surgery N Y State J Med 1986 86 651 653 3468384

[B36] Homcy CJ Southern JF Case records of the Massachusetts General Hospital. Weekly clinicopathological exercises. Case 49–1989. A 71-year-old man with thrombocytopenia and hypotension after resection of a colonic carcinoma N Engl J Med 1989 321 1595 1603 10.1056/NEJM198912073212308 2586555

[B37] Ting W Nosher JL Scholz PM Spotnitz AJ Bilateral adrenal hemorrhage after an open heart operation Ann Thorac Surg 1992 54 357 358 10.1016/0003-4975(92)91401-T 1637233

[B38] Leschi JP Goëau-Brissonnière O Coggia M Chiche L Heparin-related thrombocytopenia and adrenal hemorrhagic necrosis following aortic surgery Ann Vasc Surg 1994 8 506 508 10.1007/BF02133073 7811590

[B39] Belmore DJ Walters DN Bilateral adrenal hemorrhage following laparoscopic cholecystectomy Surg Endosc 1995 9 919 920 8525450 10.1007/BF00768894

[B40] Scheiwiller A Morel P Soravia C Bilateral adrenal gland hemorrhage after anterior deep rectum resection. Case report with review of the literature Chirurg 2002 73 628 632 10.1007/s00104-001-0403-3 12149950

[B41] Sousa Escandón A Mateos A Sánchez F González A García R Pérez J Pulpeiro JR Uribarri C Massive bilateral adrenal hemorrhage after conservative tumor surgery in the isthmus of a horseshoe kidney Actas Urol Esp 2002 26 420 424 10.1016/S0210-4806(02)72805-2 12189738

[B42] Bakaeen FG Walkes JC Reardon MJ Heparin-induced thrombocytopenia associated with bilateral adrenal hemorrhage after coronary artery bypass surgery Ann Thorac Surg 2005 79 1388 1390 10.1016/j.athoracsur.2003.09.108 15797086

[B43] Gutenberg A Lange B Gunawan B Larsen J Brück W Rohde V Verheggen R Spontaneous adrenal hemorrhage: a little-known complication of intracranial tumor surgery. Case report J Neurosurg 2007 106 1086 1088 10.3171/jns.2007.106.6.1086 17564184

[B44] Munoz Corsini L Delgado Arnaiz C Garcia Del Valle S Reboto Cortes P Lopez Del Castillo A Postoperative bilateral adrenal hemorrhage: correlation between clinical and radiological signs J Clin Anesth 2008 20 605 608 10.1016/j.jclinane.2008.05.024 19100934

[B45] Peel N Whitelaw SC Bilateral adrenal haemorrhage following right hemicolectomy Int J Colorectal Dis 2011 26 681 682 10.1007/s00384-010-1035-1 20680302

[B46] Balsach Solé A Oms Bernat LM Garrido Romero M Mato Ruiz R Sala-Pedrós J Bilateral adrenal haemorrhage after cephalic duodenopancreatectomy Cir Esp 2012 90 56 57 10.1016/j.ciresp.2010.06.014 21411065

